# Comparing the clinical outcomes of arthroereisis and osteotomy in the treatment of paediatric patients with idiopathic flexible pes planus: a systematic review and meta-analysis

**DOI:** 10.1007/s00402-026-06240-4

**Published:** 2026-02-26

**Authors:** Haoyang Chen, Hao-Xing Lai, Siddarth Venkateswaran, Andrew Kean Seng Lim, James Hoi Po Hui, Si Heng Sharon Tan

**Affiliations:** 1 Yong Loo Lin School of Medicine, National University of Singapore, Singapore, Singapore; 2 Department of Orthopaedic Surgery, National University Hospital, Singapore, Singapore

**Keywords:** Pes planus, Arthroereisis, Osteotomy, Surgical technique

## Abstract

**Introduction:**

Both subtalar arthroereisis and osteotomies are well-recognised surgical reconstructive options for paediatric pes planus deformity. We compared the clinical and radiographic outcomes of subtalar arthroereisis versus osteotomies in the surgical management of symptomatic idiopathic flexible pes planus in paediatric patients 2–18 years old. Specifically, we evaluated changes in key radiographic parameters and validated patient-reported outcome measures, as well as complications, to determine relative efficacy and safety.

**Methods:**

Electronic databases (PubMed, Embase, and The Cochrane Library) were searched from inception through August 23, 2024, following PRISMA guidelines. We reviewed studies involving patients aged 18 years or younger with idiopathic pes planus. The inclusion criteria encompassed all types of osteotomy procedures and subtalar arthroereisis, including both exosinotarsal (screw-type) and endosinotarsal (spacer-type) techniques. A random-effects meta-analysis was conducted to assess unweighted mean differences for radiographic angles and AOFAS scores.

**Results:**

Sixty studies (4,555 feet) were included: 46 arthroereisis (4,089 feet), 15 osteotomy (448 feet), and 1 combined (18 feet). Osteotomy demonstrated greater radiographic improvement in AP Meary’s angle (MD − 12.7 degrees vs. − 9.8 degrees; *p* < .0001), calcaneal pitch (MD 11.1 degrees vs. 4.1 degrees; *p* < .0001), and Kite’s angle (MD − 11.7 degrees vs. − 6.8 degrees; *p* < .0001). Arthroereisis achieved superior correction of lateral Meary’s (MD − 11.7 degrees vs. − 10.1 degrees; *p* < .0001), lateral Kite’s (MD − 7.1 degrees vs. − 4.2 degrees; *p* < .0001), and talonavicular coverage (MD − 15.6 degrees vs. − 12.7 degrees; *p* < .0001). Post-operative AOFAS improvements were similar (MD 29.2 vs. 26.4). Overall complication rates were 9.2% for arthroereisis (predominantly sinus tarsi pain) and 10.5% for osteotomy (primarily infections).

**Conclusion:**

While osteotomy yields greater correction of calcaneal inclination and hindfoot valgus, subtalar arthroereisis provides superior restoration of the lateral longitudinal arch and forefoot adduction. Despite these radiographic differences, both techniques provide equivalent functional gains. Due to its minimally invasive nature and favorable safety profile, arthroereisis is a viable first-line option, while osteotomy remains essential for correcting severe structural calcaneal pathology.

## Introduction

Pes planus, also known as flatfoot, accounts for more than 90% of clinic visits related to paediatric foot conditions [1]. While there is no objective definition of paediatric pes planus in current literature, it is recognised as a complex three-dimensional deformity characterised by hindfoot valgus, forefoot abduction, and the collapse of the medial longitudinal arch [2]. Pes planus is classified into 2 main categories: flexible flatfoot (FFF), and peroneal spastic or rigid flatfoot [3]. Typically asymptomatic, FFF comprises more than 95% of the flatfoot in infants and children and will often resolve by adolescence [4]. For symptomatic FFF patients with pain or fatigue, operative interventions are considered after the failure of conservative treatments such as corrective shoes, foot orthoses, and exercise therapies [5]. 

Soft-tissue techniques, bony procedures (osteotomies), and subtalar arthroereisis may be performed individually or in combination to re-align the foot to a functional state. Compared to open traditional surgeries, subtalar arthroereisis has been gaining increasing traction as a minimally invasive procedure to manage symptomatic FFF [6]. Through the insertion of synthetic implants into the sinus tarsi, arthroereisis seeks to stabilise the subtalar joint. This allows for the subsequent remodeling and correction of excessive forefoot abduction and subtalar eversion that underlie FFF [7]. 

Despite being associated with lower complication rates [8] and shorter hospital stays, paediatric subtalar arthroereisis continues to be a debated procedure. In a 2017 review by Bernasconi et al., [9] subtalar arthroereisis was assigned a grade C recommendation due to the poor quality of available evidence, undermining its strength of recommendation for use. Since then, there have been numerous new studies related to subtalar arthroereisis. This systematic review and meta-analysis seeks to compare arthroereisis and osteotomies for the treatment of symptomatic idiopathic flexible pes planus among the paediatric population, through the analysis of radiographic parameters and patient-reported outcome measures (PROMs).

## Methods

### Protocol registration

The study protocol was registered prospectively at the Prospero database of the University of York (registration number: CRD42024582436). The systematic review was performed using the 2020 Preferred Reporting Items for Systematic Reviews and Meta-Analyses (PRISMA) Statement [10]. 

### Search strategy

Electronic databases including PubMed, Embase and Cochrane Library were searched from inception through August 23, 2024. The keywords used were (pes planus OR pes planovalgus OR flatfoot OR flatfeet) AND (arthroereisis OR subtalar OR sinus tarsi OR osteotom*) AND (pediatric OR paediatric OR child* OR adolescen* OR young adult*). A pilot search was conducted to calibrate the inclusion criteria and refine the keyword list prior to protocol registration.

### Inclusion and exclusion criteria

All articles that reported on the clinical outcomes of arthroereisis or osteotomy in idiopathic FFF patients aged 18 and younger were reviewed. Both endosinotarsal and exosinotarsal arthroereisis were considered. Exosinotarsal screws function as impact-blocking mechanisms. The implant stem is positioned vertically within the sinus tarsi with an anterior orientation, allowing the head of the screw to make direct contact with the lateral process of the talus to restrict hyperpronation. Endosinotarsal devices are characterized as self-locking devices. They are inserted directly along the anatomical longitudinal axis of the sinus tarsi to provide structural stabilization of the subtalar joint. We also considered all types of osteotomy.

To ensure the relevance of findings to clinical practice, studies had to assess primary outcomes such as radiographic changes and validated patient reported outcomes. Non-peer-reviewed articles, editorials, opinion pieces, conference abstracts, methodological studies, scoping reviews, and literature reviews were excluded.

Furthermore, studies which included patients with accessory navicular (os tibiale externum) and Kidner procedures, talocalcaneal coalition, congenital vertical talus, skewfoot, cerebral palsy, overcorrection of talipes equinovarus, posterior tibialis insufficiency, Sever’s apophysitis, joint hyperlaxity, post-traumatic disorder, neuromuscular disease, systemic inflammatory arthropathy or other secondary pathologies were excluded.

### Data extraction, synthesis, and analysis

**Fig. 1:**
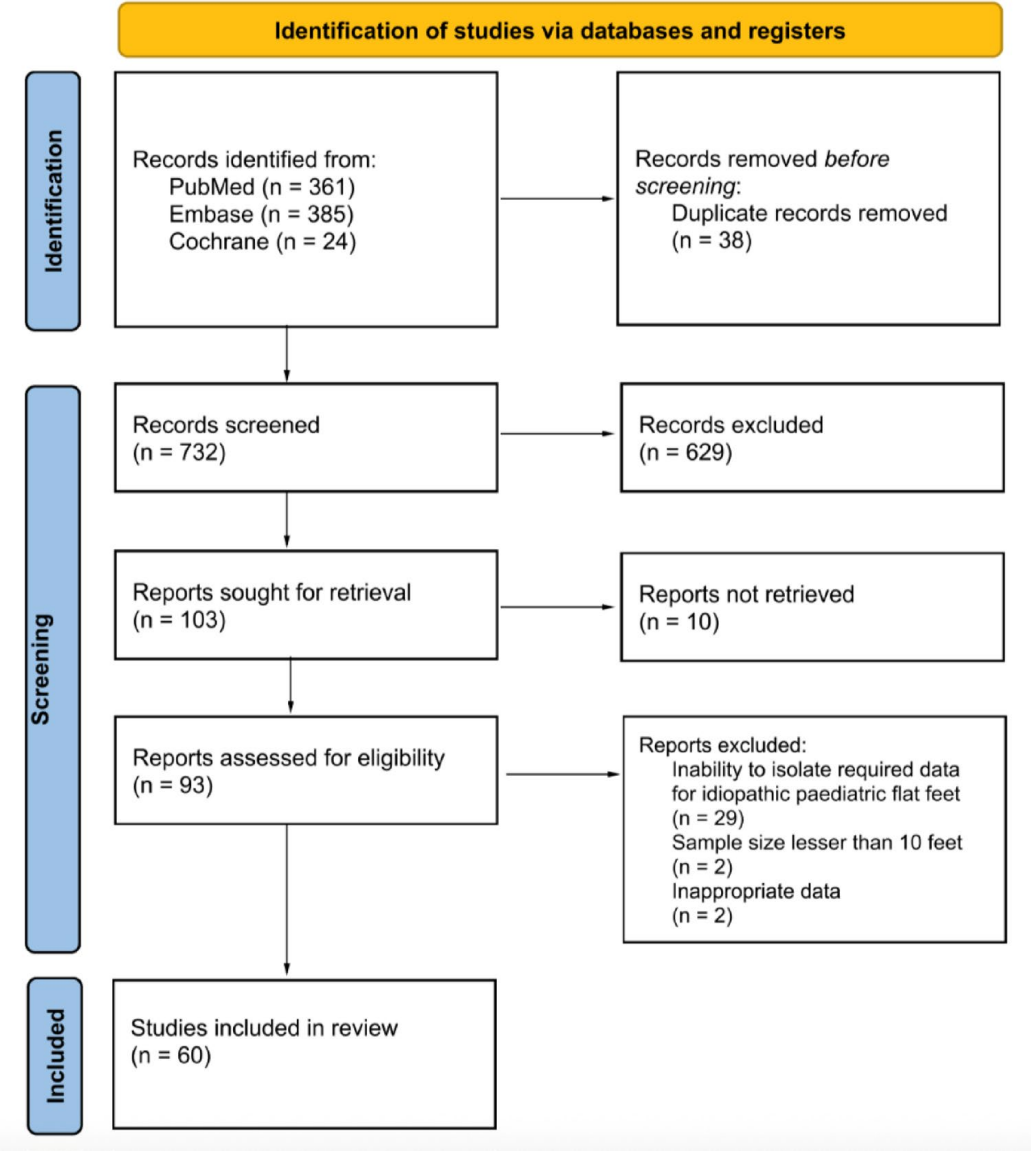
PRISMA flowchart for the systematic review detailing database search, records screened and studies included

The screening and selection process was performed independently by three authors using Rayyan (HC, HXL and SV), who initially assessed titles and abstracts for relevance after duplicates were removed. Full-text screening was conducted for studies meeting the inclusion criteria. Articles with missing data or FFF outcomes that were unable to be isolated for the paediatrics population were further excluded. The full text of the shortlisted list was also hand-searched for additional relevant studies, via forward and backward citations. Any disagreements during the screening process were resolved through discussion, and unresolved cases were adjudicated by a senior author (SHST). The study selection is summarised in Fig. 1.

We utilised a standardised data extraction form piloted on 5 randomly selected studies to ensure consistency. Data from each included study was then extracted independently by two authors (HC and HXL) in a blinded fashion. Where possible, corresponding authors of the articles were contacted to clarify any omitted data or study characteristics. Discrepancies were resolved upon discussion or revision by a third author. The study design for each paper was examined, and the levels of evidence were assigned as follows: Level I for randomised controlled trials (RCTs), Level II for cohort studies, Level III for case-controlled studies, and Level IV for case series.

To ensure meaningful comparisons, only radiological outcomes and PROMs that were reported in ten or more papers both pre and post-operatively were used for statistical analysis. Talo-first metatarsal angle refers to the angle formed by the bisection of longitudinal axes of the talus and first metatarsal [11]. Calcaneal pitch is the angle of inclination between the plantar surface of the calcaneus and the weight-bearing surface [11]. The talocalcaneal angle assesses the calcaneal alignment under the talus, and is formed by the bisection of longitudinal axes of the talus and calcaneus [12]. Another common measure evaluated was the talonavicular coverage angle, referring to the angle formed between the articular surfaces of the talus and navicular [13]. 22 studies reported on the grading of post-operative outcomes, utilising the AOFAS Ankle-Hindfoot score. Initially developed by Kitaoka et al. [14], the AOFAS Ankle-Hindfoot score has been described for use in ankle operations such as calcaneal osteotomy and subtalar arthrodesis.

A random-effect meta-analysis was conducted to compare between the pre-operative and post-operative outcomes. The random effect model supposes that the estimated effects follow a specific distribution while accounting for differences of the individual studies, under the assumption that the heterogeneity of results is due to sampling variability and real differences in the impact of treatment [15]. Heterogeneity was assessed using the Q statistic and quantified via the I² statistic [16]. Given substantial heterogeneity across studies, unweighted mean differences were calculated by subtracting the post-operative mean and the pre-operative mean to avoid over-reliance on study-specific weights that may misrepresent the overall effect under highly variable conditions. All statistical evaluations were made using Review Manager version 5.4 assuming a two-tailed test with a 95% confidence interval. P-values smaller than 0.05 were set as the threshold of statistical significance.

### Quality assessment and risk of bias (ROB)

The quality of studies was scored by two authors using the Newcastle-Ottawa Scale (NOS). The scale consists of three domains of risk of bias assessment: (i) Selection; (ii) Comparability and; (iii) Outcome.

## Results

A total of 60 studies were incorporated into this systematic review and quantitative analysis. Among the 4555 feet documented, all cases of paediatric pes planus were classified as idiopathic flexible flat feet. 46 studies (4,089 feet) employed arthroereisis, 15 studies (448 feet) employed osteotomy, while 1 study (18 feet) employed both arthroereisis and osteotomy. Most papers used calcaneal lengthening osteotomy with a few studies employing double calcaneal osteotomy or plantar and medial calcaneal displacement osteotomy. Demographics and clinical characteristics of the included studies are summarised in Table 1, while the post-operative complications of the included studies are summarised in Table 2. Mean age was 10.9 years (2–18), the few outliers at the lower age range likely represent severe, non-standard cases rather than routine practice.

**Table 1:** Demographics and clinical characteristics of the studies included in the systematic review

Reference	Level of evidence	No. of patients	No. of feet	Mean age (years)^	Gender	Procedure	Additional procedure	Type of implant	Mean follow-up (months)^
Abhishek et al. [75]	3	65	102	12.8 (8–18)	M: 64* F: 38 *Reported by feet	Calcaneal lengthening osteotomy	Achilles tendons lengthening, gastrocnemius recession, medial cuneiform osteotomy	NA	NR
Abubeih et al. [64]	4	26	45	12.1 (7–14)	M: 9 F: 17	Arthroereisis	NIL	Screw (Calcaneo-Stop)	35.3 (30–40)
Alvarez et al. [18]	4	40	79	10 (5–17)	M: 17 F: 23	Arthroereisis	Achilles tendon lengthening	Screw (Calcaneo-Stop)	47 (12–88)
Baghdadi et al. [65]	4	20	30	10.4 (8–12.5)	M: 12 F: 8	Calcaneal lengthening osteotomy	Achilles tendon lengthening	NA	23.1 (9–50)
Bai et al. [19]	4	25	45	11.2 (9–15)	M: 17 F: 8	Arthroereisis	Gastrocnemius recession	Spacer	28.5 (24–55)
Bernasconi et al. [20]	3	31	62	10.5 (8–15)	M: 22 F: 9	Arthroereisis	NIL	Screw	62
Bittar et al. [21]	2	20	23	8.13 (5–14)	NR	Arthroereisis	NIL	Screw	33 (24–43)
Bobinski et al. [22]	2	27	35	10.5 (7–14)	M: 16 F: 11	Arthroereisis	Achilles tendon lengthening	Screw (Spherus)	14.76 (12–26)
Bruyn et al. [63]	4	14	18	12.1 (8–18)	NR	Calcaneal lengthening osteotomy and subtalar arthrorereisis	Achilles tendon lengthening, plantarflexory medial cuneiform osteotomy, Cotton osteotomy	Spacer (STA-Peg)	25.6 (6–54)
Calvo et al. [24]	4	52	103	11.6 (7.11–14.8)	M: 32 F: 20	Arthroereisis	NIL	Screw	187.9 (163.3–212.6)
Caravaggi et al. [25]	2	13	26	11.3	NR	Arthroereisis	NIL	Spacer for left foot, screw for right foot	12.5
Chong et al. [26]	2	7	13	12.8 (8–17)* * Reported combined age of patients undergoing arthroereisis and calcaneal lengthening osteotomy	NR	Arthroereisis	NIL	Screw (Vilex)	12.7
Chong et al. [26]	2	8	11	12.8 (8–17)* * Reported combined age of patients undergoing arthroereisis and calcaneal lengthening osteotomy	NR	Calcaneal lengthening osteotomy	Gastrocnemius recession, bilateral peroneal tendon transfer, calcaneocuboid fusion	NA	12.7
Cicchinelli et al. [27]	4	20	28	11.6 (4–16)	M: 11 F: 9	Arthroereisis	Gastrocnemius recession, Cotton osteotomy, Hooke arthrodesis	Spacer (Maxwell Brancheau)	9 (0.8–32.8)
Das et al. [28]	2	15	25	12.5	M: 10 F: 5	Arthroereisis	Achilles tendon lengthening	Screw (Calcaneo-Stop)	54 (32–75)
de Bot et al. [23]	4	16	26	12.5 (10–15)	M: 6 F: 10	Arthroereisis	Achilles tendon lengthening, spring ligament reconstruction	Spacer (Kalix II)	47 (19–79)
DeFrancesco et al. [17]	2	19	25	13.8 (10.3–16.5)	M: 11* F: 14 * Reported by feet	Calcaneal lengthening osteotomy	Achilles tendon lengthening, gastronecmius recession, medial cuneiform plantarflexion osteotomy, medialising calcaneal osteotomy	NA	12.3 (2.3–24.5)
El-Tayeby et al. [66]	4	11	19	10.7 (9–14)	M: 4 F: 7	Calcaneal lengthening osteotomy	Achilles tendon lengthening, tibialis anterior tendon transfer, naviculocuneiform joint arthrodesis	NA	29 (8–42)
Elmarghany et al. [29]	4	42	84	9.9 (7–15)	M: 26 F: 16	Arthroereisis	NIL	Screw (Asnis III)	29.1 (2–48)
Eysel et al. [30]	3	18	25	12.5 (10–16)	M: 14 F: 11	Arthroereisis	NIL	Screw (ProStop)	46.8 (4.8–96)
Garcia et al. [31]	4	14	24	12 (9–14)	M: 7 F: 7	Arthroereisis	NIL	Spacer (Maxwell Brancheau)	68.3 (24–105)
Ghaznavi et al. [33]	4	44	57	10.2 (5–15)	M: 27 F: 17	Arthroereisis	Achilles tendon lengthening	Screw (Calcaneo-Stop)	NR
Ghaznavi et al. [76]	4	50	50	9.2	M: 27 F: 23	Calcaneal lengthening osteotomy	Achilles tendon lengthening, tibialis posterior tendon transfer	NA	31.2
Giannini et al. [32]	4	44	88	11.7 (8–14)	M: 31 F: 13	Arthroereisis	Achilles tendon lengthening	Screw (Calcaneo-Stop)	56 (50–63)
Gutierrez et al. [34]	4	37	65	9.4 (5–14)	M: 22 F: 15	Arthroereisis	Achilles tendon lengthening	Spacer (Giannini)	26.5 (13–51)
Herdea et al. [35]	3	33	66	19.9	M: 19 F: 14	Arthroereisis	Achilles tendon lengthening	Spacer	24
Hosny et al. [69]	4	19	28	10 (6–15)	M: 10 F: 9	Calcaneal lengthening osteotomy	Achilles tendon lengthening	NA	10.3 (8–16)
Hsieh et al. [36]	4	102	204	9 (7–11)	M: 72 F: 30	Arthroereisis	Gastrocnemius recession	Spacer (Bioarch)	24
Indino et al. [37]	4	56	112	NR (9–14)	M: 34 F: 22	Arthroereisis	NIL	Spacer (Conical)	40.1 (18–112.4)
Jay et al. [38]	4	20	34	10.6 (4–17)	M: 13 F: 7	Arthroereisis	Gastrocnemius recession	Spacer	18.4 (6–34)
Jerosch et al. [39]	4	18	21	11.9 (8–14)	M: 13 F: 5	Arthroereisis	Gastrocnemius recession	Screw	32.4 (6–84)
Kellermann et al. [40]	4	25	43	10 (7–14)	M: 18 F: 7	Arthroereisis	NIL	Screw (Calcaneo-Stop)	9.7 (3–19)
Koning et al. [41]	4	27	54	8 (4–11)	M: 22 F: 5	Arthroereisis	NIL	Spacer	151.2 (12.6–193.2)
Kubo et al. [42]	4	50	95	11.3 (5–15)	NR	Arthroereisis	Achilles tendon lengthening, gastrocnemius recession	Screw	35.8 (13–79)
Kubo et al. [43]	4	14	19	9.3 (5–13)	M: 13 F: 6	Arthroereisis	Achilles tendon lengthening, gastrocnemius recession	Screw	39.4
Lai et al. [67]	4	13	23	12.3 (11–16)	NR	Double calcaneal osteotomy	Gastronecmius recession	NA	49.7 (30.9–73.4)
Le Gall et al. [44]	4	48	78	11.3 (7–16)	NR	Arthroereisis	NIL	Spacer	35 (18–84)
Li et al. [45]	4	32	32	9.5 (8–12)	M: 18 F: 12	Arthroereisis	Achilles tendon lengthening	Spacer (Talar-Fit)	25.3 (18–36)
Luna et al. [68]	4	14	26	12.8 (11–14.6)	M: 8 F: 6	Double calcaneal osteotomy	Achilles tendon lengthening	NA	91
Mazzotti et al. [46]	4	34	64	12 (9.2–14.9)	M: 21 F: 13	Arthroereisis	Achilles tendon lengthening	Spacer (BFFI)	180 (120–140)
Megremis et al. [47]	4	14	28	10.7 (8–14)	M: 10 F: 4	Arthroereisis	Achilles tendon lengthening	Spacer (Maxwell Brancheau)	35.1 (19–60)
Memeo et al. [48]	2	NR	402	NR (8–16)	NR	Arthroereisis	Achilles tendon lengthening	Spacer for 200 feet, screw for 202 feet	130 (35–150)
Morsy et al. [49]	2	19	30	11.8 (6–16)	M: 9 F: 10	Arthroereisis	Achilles tendon lengthening, Gastronecmius recession	Spacer	18
Novillo et al. [50]	4	86	134	10.3 (2–15)	M: 45 F: 41	Arthroereisis	NIL	Spacer (HyProCure)	3
Papamerkouriou et al. [51]	2	6	12	11.1 (15.5–17.5)	NR	Arthroereisis	NIL	Spacer (Kalix II)	NR
Pavone et al. [52]	4	68	136	12.7 (9–15)	M: 38 F: 30	Arthroereisis	NIL	Screw (Calcaneo-Stop)	57.6 (15–96)
Pavone et al. [53]	4	242	410	11(7–14)	M: 157 F: 85	Arthroereisis	Achilles tendon lengthening	Screw (Calcaneo-Stop)	88 (14–157)
Pellegrin et al. [54]	4	485	732	11.5 (5–17.9)	M: 267 F: 218	Arthroereisis	NIL	Screw	54 (37.2–158.4)
Riva et al. [55]	4	62	124	12.1 (10.5–14.5)	M: 42 F: 20	Arthroereisis	NIL	Spacer (PitStop)	12.7 (4.56–27.5)
Roth et al. [56]	4	48	94	11.4 (8–14)	M: 17 F: 31	Arthroereisis	NIL	Screw (Calcaneo-Stop)	20 (12–112)
Ruiz-Picazo et al. [57]	4	16	32	9 (7–11)	M: 13 F: 3	Arthroereisis	NIL	Spacer	NR
Sakr et al. [70]	4	16	28	11.6 (9–15)	M: 8 F: 8	Calcaneal lengthening osteotomy	Gastrocnemius recession, tibialis anterior tendon rerouting	NA	38.9 (24–60)
Silva et al. [71]	4	23	30	11.5 (6.9–16.1)	M: 13 F: 10	Plantar and medial calcaneal displacement osteotomy with opening wedge cuboid osteotomy	NIL	NA	37 (26–60)
Szesz et al. [58]	2	30	41	10 (6–16)	NR	Arthroereisis	NIL	Spacer	8 (6–12)
Tahririan et al. [59]	1	35	35	10.1 (6.7–13.4)	M: 23 F: 12	Arthroereisis	Gastrosoleus recession	Screw (Calcaneo-Stop)	17.6 (11–20)
Tahririan et al. [59]	1	31	31	10.2 (7.11–13.3)	M: 19 F: 12	Calcaneal lengthening osteotomy	Gastrosoleus recession	NA	17.6 (11–20)
Vogt et al. [60]	2	73	113	10.8 (5–16)	M: 45 F: 28	Arthroereisis	Achilles tendon lengthening, gastrocnemius recession	Spacer (Kalix) for 21 feet, spacer (Giannini) for 56 feet, screw for 36 feet	29 (1–111)
Wang et al. [61]	4	NR	22	13* * Includes patients with accessory navicular	NR	Arthroereisis	Achilles tendon lengthening, gastrocnemius recession, flexor digitorum longus transfer, Cotton osteotomy, lateral column lengthening, medial displacement calcaneal osteotomy	Spacer (Talar-Fit)	32.8 (10–71)* * Includes patients with accessory navicular
Xu et al. [72]	4	13	15	15.2 (10–18)	M: 9 F: 4	Double calcaneal osteotomy	Achilles tendon lengthening, gastrocnemius recession, Cotton osteotomy,	NA	34.5 (21–60)
Zaghloul et al. [73]	4	12	15	10.8 (8–13.5)	M: 6 F: 9	Calcaneal lengthening osteotomy	Achilles tendon lengthening, tibialis posterior tendon advancement	NA	15.2 (12–18)
Zahid et al. [62]	4	30	60	9.5 (5–15)	M: 21 F: 9	Arthroereisis	NIL	Spacer for 15 feet, screw for 15 feet	18
Zairi et al. [74]	4	12	15	12 (10–16)	M: 7 F: 5	Calcaneal lengthening osteotomy	NIL	NA	18 (12–36)

^Values are presented as mean (range)

**Table 2:** Postoperative complications of the studies included in the systematic review

Reference	No. of feet with complication	Premature implant removal	Persistent pain	Need for revision surgery	Other complications
Abhishek et al	14	NIL	10 cases	10 cases	Superficial infection (2 cases)
					Deep infection (2 cases)
Abubeih et al	0	NIL	NIL	NIL	NIL
Alvarez et al	11	NIL	NIL	5 cases	Screw breakage (5 cases)
					Superficial infection (2 cases)
					Implant osteolysis (1 case)
					Plantar protrusion of screw (2 cases)
Baghdadi et al	5	NIL	NIL	NIL	Skin and peroneal tendon irritation (3 cases)
					Distal segment displacement (2 cases)
Bai et al	2	NIL	2 cases	NIL	NIL
Bernasconi et al	14	14 cases	14 cases	NIL	NIL
Bittar et al	2	2 cases	NIL	NIL	Implant loosening (2 cases)
Bobinski et al	3	NIL	3 cases	NIL	NIL
Bruyn et al	2	NIL	NIL	NIL	Undercorrection (2 cases)
Calvo et al	10	NIL	NIL	10 cases	Implant dislocation (4 cases)
					Superficial infection (1 case)
Caravaggi et al	NR	NR	NR	NR	NR
Chong et al	2	2 cases	2 cases	NIL	NIL
Chong et al	2	1 case	NIL	NIL	Partial exclusion of graft and staple (1 case)
					Wound dehiscence (1 case)
Cicchinelli et al	NR	NR	NR	NR	NR
Das et al	4	1 case	1 case	1 case	Implant loosening (1 case)
					Delayed wound healing and contracture of the peroneal muscles (3 cases)
de Bot et al	6	NIL	6 cases	6 cases	Implant migration (6 cases)
DeFrancesco et al	6	1 case	2 cases	1 case	Second metatarsalgia (1 case)
					Saphenous nerve palsy due to regional anesthesia (2 cases)
					Calcaneocuboid joint arthritis (1 case)
					Peroneal tendon irritation (1 case)
					Residual hindfoot valgus (1 case)
El-Tayeby et al	2	NIL	NIL	NIL	Naviculocuneiform pseudarthrosis (2 cases)
Elmarghany et al	3	1 case	NIL	NIL	Screw synovitis (1 case)
					Sunken screw (1 case)
					Deformity undercorrection (1 case)
Eysel et al	NR	NR	NR	NR	NR
Garcia et al	1	NIL	1 case	NIL	NIL
Ghaznavi et al	2	2 cases	2 cases	NIL	NIL
Ghaznavi et al	1	NIL	NIL	NIL	Superficial surgical site infection (1 case)
					Undercorrection (7 cases)
					Overcorrection (1 case)
Giannini et al	2	2 cases	NIL	NIL	Implant breakage (2 cases)
Gutierrez et al	6	3 cases	4 cases	1 cases	External tibiotarsal sprain (2 cases)
Herdea et al	NR	NR	NR	NR	NR
Hosny et al	1	NIL	NIL	NIL	Superficial surgical site infection (1 case)
Hsieh et al	86	NR	NR	NR	Implant extrusion (86 cases)
Indino et al	0	NIL	NIL	NIL	NIL
Jay et al	10	NIL	9 cases	NIL	Significant limp (1 case)
Jerosch et al	0	NA	NA	NA	NA
Kellermann et al	0	NIL	NIL	NIL	NIL
Koning et al	2	1 case	NIL	1 case	Implant dislocation (2 cases)
Kubo et al	0	NIL	NIL	NIL	NIL
Kubo et al	0	NIL	NIL	NIL	NIL
Lai et al	2	NIL	NIL	NIL	Asymptomatic nonunion (1 case)
					Superficial infection (1 case)
Le Gall et al	11	5 cases	NIL	NIL	Spontaneous screw expulsion (6 cases)
Li et al	0	NIL	NIL	NIL	NIL
Luna et al	3	NIL	NIL	NIL	Calcanoeocuboid joint subluxation (3 cases)
Mazzotti et al	0	NIL	NIL	NIL	NIL
Megremis et al	0	NIL	NIL	NIL	NIL
Memeo et al	57	14 cases	20 cases	NIL	Incomplete correction (23 cases)
Morsy et al	1	1 case	1 case	1 case	Implant extrusion (1 case)
Novillo et al	8	3 cases	2 cases	1 case	Ankle effusion (3 cases)
					Implant migration (1 case)
					Peroneal muscle contractures (2 cases)
					4th metatarsal stress fracture (2 cases)
Papamerkouriou et al	0	NIL	NIL	NIL	NIL
Pavone et al	12	4 cases	4 cases	NIL	Local symptoms at incision (4 cases)
					Screw loosening (3 cases)
					Screw breakage (1 case)
					Superficial infection (4 cases)
Pavone et al	20	NIL	9 cases	NIL	Local symptoms at incision (10 cases)
					Peroneal muscle contractures (3 cases)
					Superficial infection (7 cases)
Pellegrin et al	25	8 cases	NIL	2 cases	4th metatarsal stress fracture (3 cases)
					Painful contracture of peroneal muscles (14 cases)
					Ankle joint effusion or haemarthrosis (8 cases)
Riva et al	1	NIL	NIL	NIL	Wound dehiscence (1 case)
Roth et al	11	2 cases	2 cases	2 cases	Screw breakage (9 cases)
					Incorrect screw position (2 cases)
Ruiz-Picazo et al	4	NIL	NIL	NIL	Overcorrection of the foot and expulsion of implant (4 cases)
Sakr et al	8	NIL	NIL	NIL	Superficial wound infection (3 cases)
					Hardware irritation (5 cases)
Silva et al	4	NIL	NIL	NIL	Superficial infection (1 case)
					Suture dehiscence (1 case)
					K-wire path infection (2 cases)
Szesz et al	7	NIL	6 cases	1 case	Undercorrection (1 case)
Tahririan et al	1	1 case	1 case	NIL	NIL
Tahririan et al	1	NIL	1 case	NIL	Graft displacement (1 case)
Vogt et al	29	14 cases	11 cases	NIL	Peroneal muscle contractures (4 cases)
Wang et al	7	1 case* * The study by Wang et al. was not included in the tabulation of overall complication rate since it was not possible to isolate the specific complications from FFF patients who did not have an accessory navicular	6 cases* * The study by Wang et al. was not included in the tabulation of overall complication rate since it was not possible to isolate the specific complications from FFF patients who did not have an accessory navicular	NIL	Implant dislocation due to fall (1 case)* * The study by Wang et al. was not included in the tabulation of overall complication rate since it was not possible to isolate the specific complications from FFF patients who did not have an accessory navicular
Xu et al	1	NIL	NIL	NIL	Superficial infection (1 case)
Zaghloul et al	0	NIL	NIL	NIL	NIL
Zahid et al	3	NIL	1 case	NIL	Talar osteolysis (2 cases)
					Aseptic loosening and peri-screw fracture (1 case)
Zairi et al	5	NIL	NIL	NIL	Superficial surgical site infection (2 cases)
					Inflammatory skin granuloma (3 cases)

A large number of radiographic parameters and PROMs were described across the selected studies. Pre and post-operative mean outcomes and the unweighted mean difference (MD) are presented in Table 3, alongside the 95% confidence interval (CI).

**Table 3:** Pre and post-operative mean of various radiographic and patient reported outcomes

Outcome	Pre-operative mean (95% CI)	Post-operative mean (95% CI)	Unweighted mean difference (95% CI)
Lateral talo-first metatarsal angle (Meary’s angle)	Arthroereisis: 17.60 (8.68)	Arthroereisis: 5.89 (5.92)	Arthroereisis: −11.71 [−11.84, −11.58]
	Osteotomy: 18.74 (8.28)	Osteotomy: 8.66 (10.73)	Osteotomy: −10.08 [−10.34, −9.82]
	Arthroereisis and osteotomy: 20.20 (9.00)	Arthroereisis and osteotomy: 6.50 (9.90)	Arthroereisis and osteotomy: −13.70 [−14.12, −13.28]
			**P-value: < 0.0001**
Anteriorposterior talo-first metatarsal angle	Arthroereisis: 18.30 (11.24)	Arthroereisis: 8.48 (6.87)	Arthroereisis: −9.82 [−10.24, −9.40]
	Osteotomy: 21.01 (11.33)	Osteotomy: 8.30 (6.13)	Osteotomy: −12.71 [−13.29, −12.13]
	Arthroereisis and osteotomy: 17.30 (11.00)	Arthroereisis and osteotomy: 1.20 (10.20)	Arthroereisis and osteotomy: −16.10 [−16.47, −15.73]
			**P-value: < 0.0001**
Calcaneal pitch	Arthroereisis: 11.96 (5.31)	Arthroereisis: 16.01 (5.37)	Arthroereisis: 4.05 [4.05, 4.05]
	Osteotomy: 10.32 (5.64)	Osteotomy: 21.46 (6.16)	Osteotomy: 11.14 [11.09, 11.19]
	Arthroereisis and osteotomy: 8.20 (4.10)	Arthroereisis and osteotomy: 16.80 (5.10)	Arthroereisis and osteotomy: 8.60 [8.14, 9.06]
			**P-value: < 0.0001**
Anterior–posterior talocalcaneal angle (Kite’s angle)	Arthroereisis: 29.53 (7.09)	Arthroereisis: 22.72 (6.43)	Arthroereisis: −6.81 [−6.85, −6.77]
	Osteotomy: 35.28 (5.24)	Osteotomy: 23.55 (4.95)	Osteotomy: −11.73 [−11.79, −11.67]
	Arthroereisis and osteotomy: 26.00 (4.00)	Arthroereisis and osteotomy: 16.50 (4.50)	Arthroereisis and osteotomy: −9.50 [−9.73, −9.27]
			**P-value: < 0.0001**
Lateral talocalcaneal angle	Arthroereisis: 36.93 (9.42)	Arthroereisis: 29.79 (10.91)	Arthroereisis: −7.14 [−7.21, −7.07]
	Osteotomy: 26.57 (17.9)	Osteotomy: 22.33 (22.3)	Osteotomy: −4.24 [−4.88, −3.60]
	Arthroereisis and osteotomy: 40.40 (8.20)	Arthroereisis and osteotomy: 35.47 (7.35)	Arthroereisis and osteotomy: −4.93 [−5.32, −4.54]
			**P-value: < 0.0001**
Talonavicular coverage angle	Arthroereisis: 25.76 (11.15)	Arthroereisis: 10.13 (8.15)	Arthroereisis: −15.63 [−15.87, −15.39]
	Osteotomy: 22.99 (12.44)	Osteotomy: 10.27 (9.30)	Osteotomy: −12.72 [−13.22, −12.22]
	Arthroereisis and osteotomy: 24.30 (10.40)	Arthroereisis and osteotomy: 7.05 (7.40)	Arthroereisis and osteotomy: −17.25 [−8.64, −5.86]
			**P-value: < 0.0001**
AOFAS Ankle-Hindfoot score	Arthroereisis: 66.19 (15.31)	Arthroereisis: 92.56 (9.51)	Arthroereisis: 26.37 [25.94, 26.80]
	Osteotomy: 59.82 (11.42)	Osteotomy: 89.00 (7.67)	Osteotomy: 29.18 [28.60, 29.77]
	Arthroereisis and osteotomy: NA	Arthroereisis and osteotomy: NA	Arthroereisis and osteotomy: NA

Significant P-values are bolded

**Table 4:** Newcastle-Ottawa Scale (NOS) of included studies

Reference	Selection	Comparability	Outcome	Total Score	ROB
Abhishek et al.	★★★★	★	★	6	Moderate
Abubeih et al.	★★★		★★	5	Moderate
Alvarez et al.	★★★		★★	5	Moderate
Baghdadi et al.	★★★		★★	5	Moderate
Bai et al.	★★★		★★★	6	Moderate
Bernasconi et al.	★★★★	★	★★★	8	Low
Bittar et al.	★★★		★★★	6	Moderate
Bobinski et al.	★★★		★★	5	Moderate
Bruyn et al.	★★★		★★★	6	Moderate
Calvo et al.	★★★		★★	5	Moderate
Caravaggi et al.	★★★★	★	★★	7	Low
Chong et al.	★★★★	★	★★★	8	Low
Cicchinelli et al.	★★★		★★	5	Moderate
Das et al.	★★★		★★★	6	Moderate
de Bot et al.	★★★		★★★	6	Moderate
DeFrancesco et al.	★★★		★★	5	Moderate
El-Tayebey et al.	★★★		★★★	6	Moderate
Elmarghany et al.	★★★		★★★	6	Moderate
Eysel et al.	★★★★	★	★★★	8	Low
Garcia et al.	★★★		★★	5	Moderate
Ghaznavi et al.	★★★		★	4	Moderate
Ghaznavi et al.	★★★		★★	5	Moderate
Giannini et al.	★★★		★★★	6	Moderate
Gutierrez et al.	★★★		★★★	6	Moderate
Herdea et al.	★★★★	★	★★	7	Low
Hosny et al.	★★★		★★	5	Moderate
Hsieh et al.	★★★★	★	★★	7	Low
Indino et al.	★★★		★★★	6	Moderate
Jay et al.	★★★		★★	5	Moderate
Jerosch et al.	★★★		★★★	6	Moderate
Kellermann et al.	★★★		★	4	Moderate
Koning et al.	★★★		★★	5	Moderate
Kubo et al.	★★★	★	★	5	Moderate
Kubo et al.	★★★		★★★	6	Moderate
Lai et al.	★★★		★★★	6	Moderate
Le Gall et al.	★★★		★★★	6	Moderate
Li et al.	★★★		★★	5	Moderate
Luna et al.	★★★		★★★	6	Moderate
Mazzotti et al.	★★★		★★★	6	Moderate
Megremis et al.	★★★		★★★	6	Moderate
Memeo et al.	★★★★	★	★★★	8	Low
Morsy et al.	★★★		★★	5	Moderate
Novillo et al.	★★★		★★★	6	Moderate
Papamerkouriou et al.	★★★		★★	5	Moderate
Pavone et al.	★★★		★★★	6	Moderate
Pavone et al.	★★★		★★★	6	Moderate
Pellegrin et al.	★★★		★★★	6	Moderate
Riva et al.	★★★		★★	5	Moderate
Roth et al.	★★★		★★★	6	Moderate
Ruiz-Picazo et al.	★★★		★★★	6	Moderate
Sakr et al.	★★★		★★★	6	Moderate
Silva et al.	★★★		★★★	6	Moderate
Szesz et al.	★★★		★★	5	Moderate
Tahririan et al.	★★★	★	★★	6	Moderate
Vogt et al.	★★★★	★	★★★	8	Low
Wang et al.	★★★	★	★★★	7	Low
Xu et al.	★★★		★★★	6	Moderate
Zaghloul et al.	★★★		★★	5	Moderate
Zahid et al.	★★★★	★	★★★	8	Low
Zairi et al.	★★★		★★★	6	Moderate

*Low ROB: 7–9 stars; Moderate ROB: 4–6 stars; High ROB: 0–3 stars. A maximum of 4 stars for Selection (1 each for representativeness of the exposed cohort, selection of the non-exposed cohort, ascertainment of exposure, and demonstration that the outcome was not present at the start of the study), 2 stars for Comparability (controlling for confounding factors), and 3 stars for Outcome (1 each for assessment of outcome, whether follow-up was long enough for outcomes to occur, and the adequacy of follow-up)

**Fig. 2:**
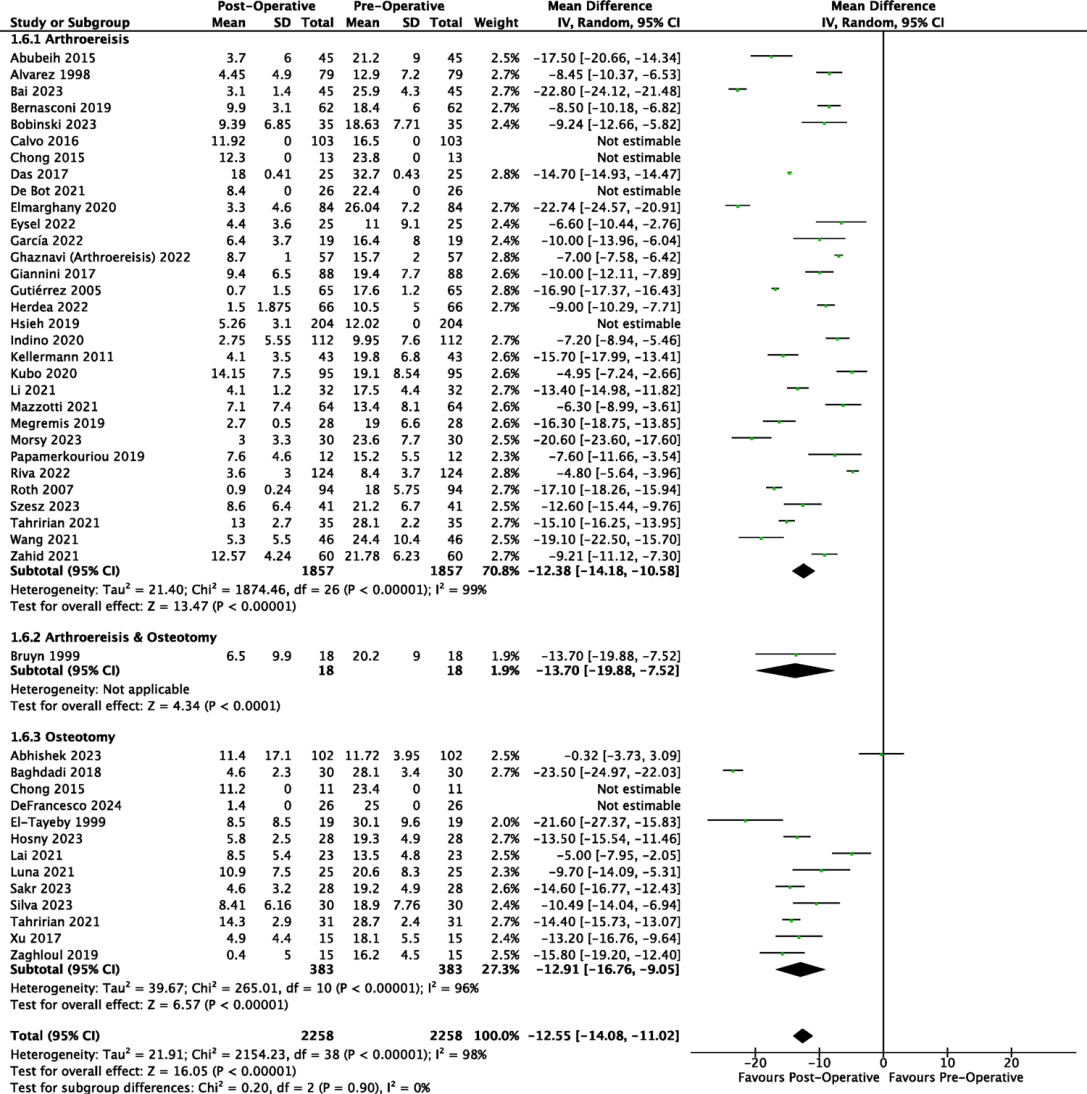
Forest plot comparing the pre- and post-operative outcomes of the lateral talo-first metatarsal angle (Meary’s angle)

**Fig. 3:**
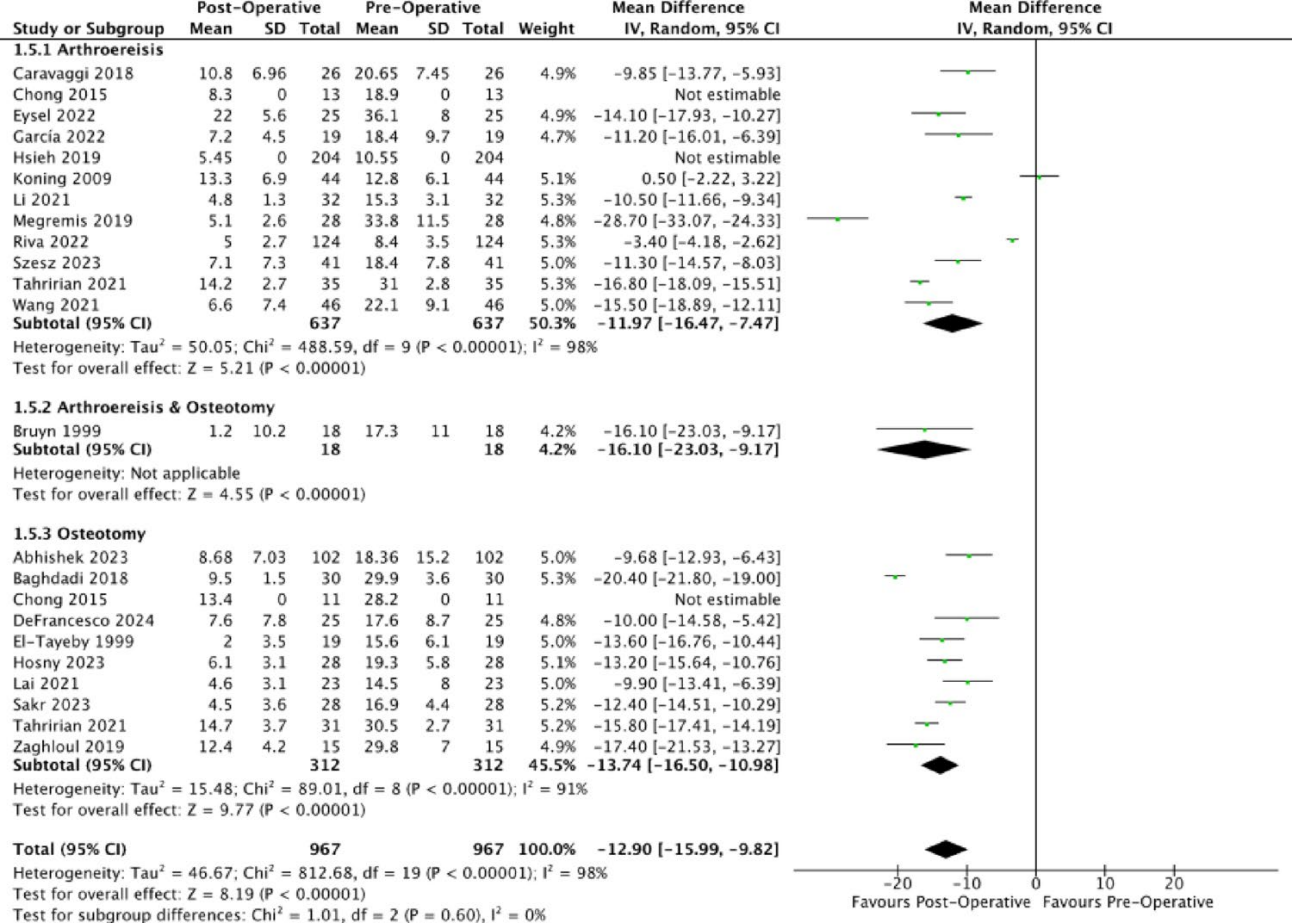
Forest plot comparing the pre- and post-operative outcomes of the anterior-posterior talo-first metatarsal angle

All analysed publications reported a decrease in talo-first metatarsal angle post-operatively from both the AP and lateral views (Figs. 2 and 3). The unweighted MDs of the lateral talo-first metatarsal angle for arthroereisis, osteotomy and combined operations respectively were − 11.71 degrees (95% CI: −11.84, −11.58); −10.08 degrees (95% CI: −10.34, −9.82); −13.70 degrees (95% CI: −14.12, −13.28). The corresponding post-operative lateral talo-first metatarsal angles of 5.89 degrees, 8.66 degrees and 6.50 degrees were within that of the normal population’s mean (normal: 0.0–13.0) [77], indicative of a reduction in the severity of the collapse. Arthroereisis demonstrated a significantly greater reduction in the lateral talo-first metatarsal angle (*p* <.0001) compared to osteotomy.

The unweighted MDs of the anteroposterior talo-first metatarsal angle for arthroereisis, osteotomy and combined operations respectively were − 9.82 degrees (95% CI: −10.24, −9.40); −12.71 degrees (95% CI: −13.29, −12.13); −16.10 degrees (95% CI: −16.47, −15.73). The corresponding post-operative anteroposterior talo-first metatarsal angles of 8.48 degrees, 8.30 degrees and 1.20 degrees were within that of the normal population’s mean (normal: 0.0–10.0) [78], indicative of a reduction in the severity of the collapse of the medial longitudinal arch. Osteotomy demonstrated a significantly greater reduction in the anteroposterior talo-first metatarsal angle (*p* <.0001) compared to subtalar arthroereisis.

**Fig. 4:**
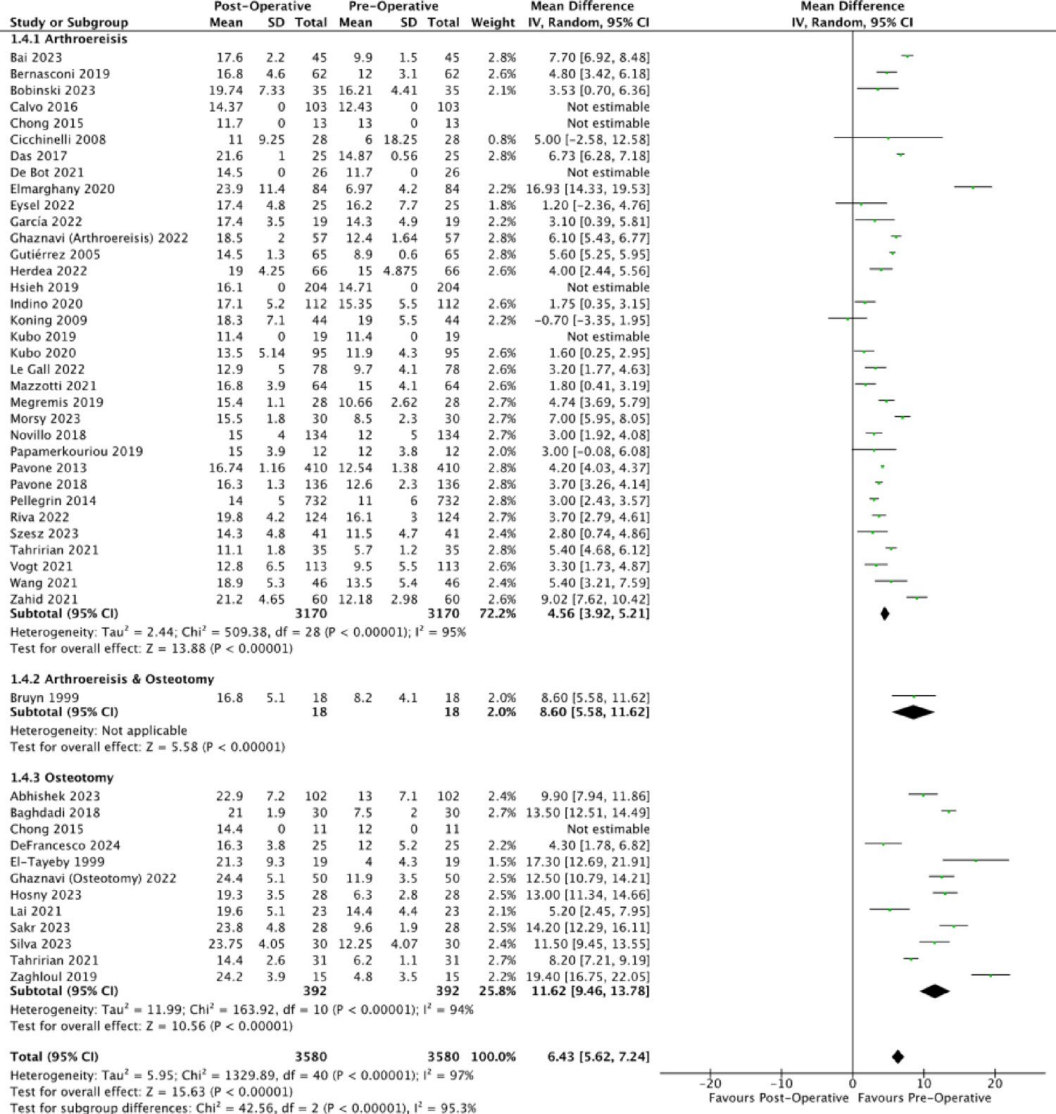
Forest plot comparing the pre- and post-operative outcomes of the calcaneal pitch

Regarding the calcaneal pitch, analysed papers consistently reported an increase in calcaneal pitch with unweighted MDs of (4.05 degrees (95% CI: 4.05, 4.05), 11.14 degrees (95% CI: 11.09, 11.19), 8.60 degrees (95% CI: 8.14, 9.06)) for arthroereisis, osteotomy and combined operations (Fig. 4). The corresponding mean post-operative calcaneal pitches were 16.01 degrees, 21.46 degrees and 16.80 degrees, lying close to the normal range of 18.0 degrees to 30.0 degrees [79]. Compared to subtalar arthroereisis, osteotomy demonstrated a significantly greater improvement in calcaneal pitch (*p* <.0001).

**Fig. 5:**
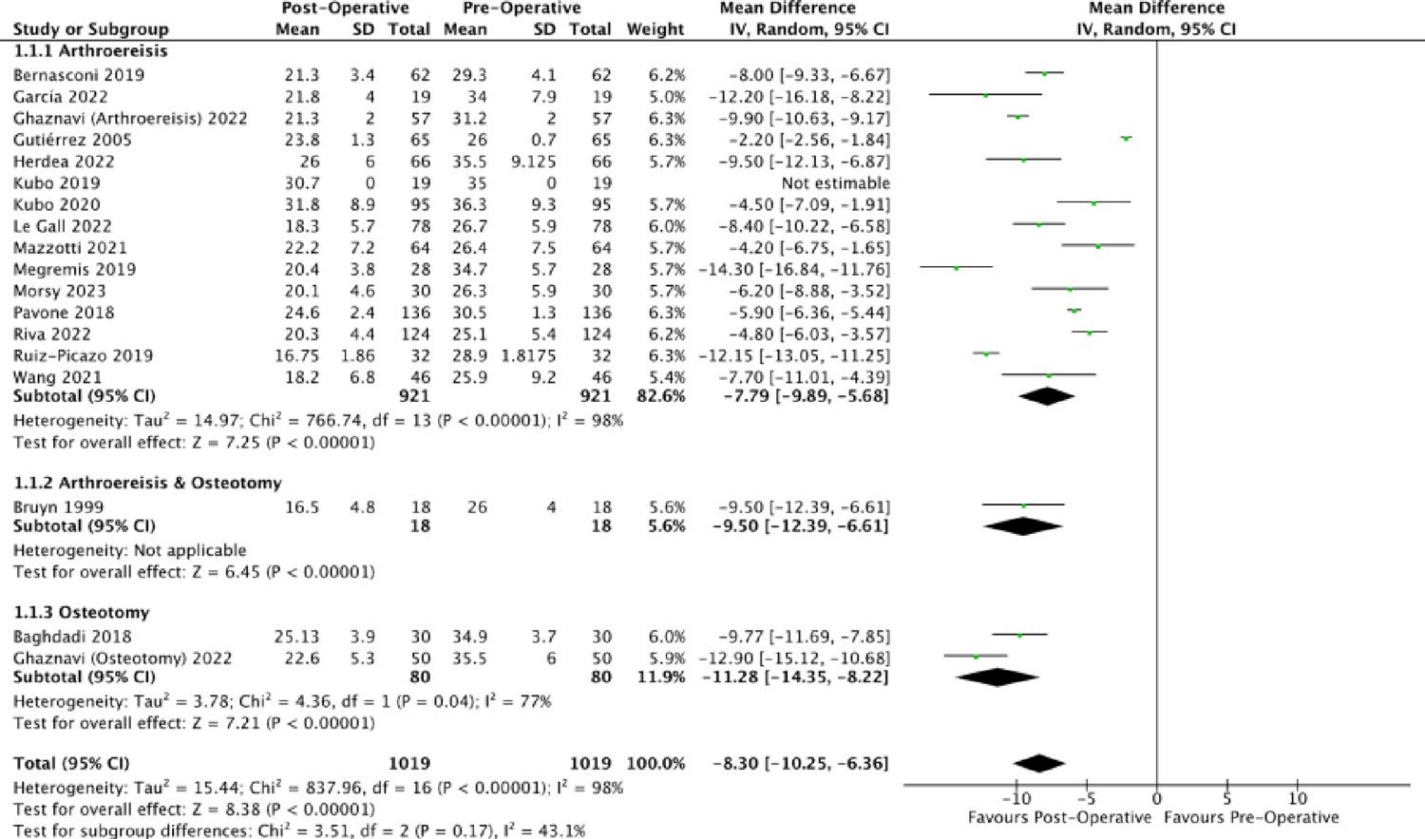
Forest plot comparing the pre- and post-operative outcomes of the anterior-posterior talocalcaneal angle (Kite’s angle)

**Fig. 6:**
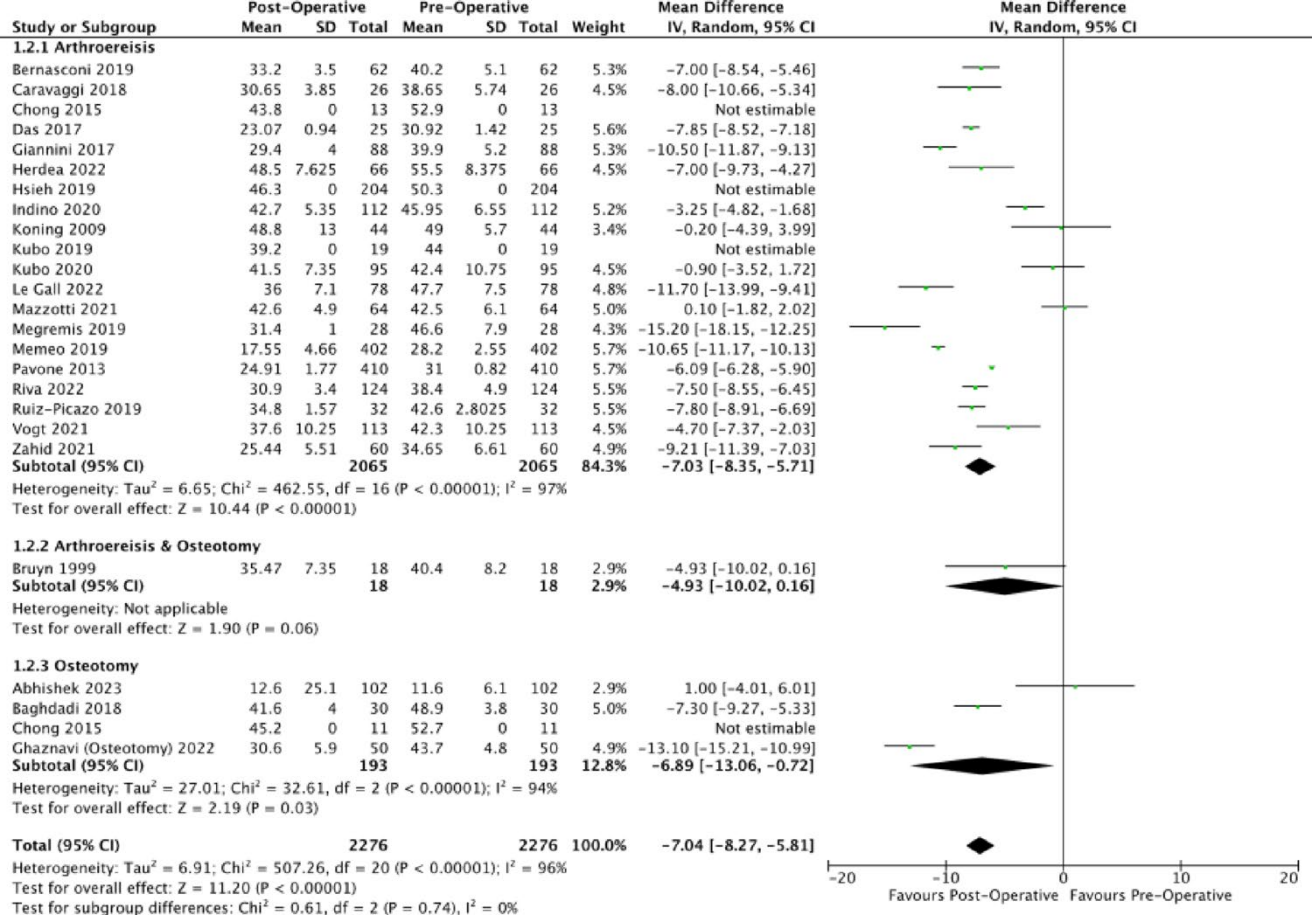
Forest plot comparing the pre- and post-operative outcomes of the lateral talocalcaneal angle

In addition to the radiological outcomes which examined the medial longitudinal arch, the talocalcaneal angle was also reported from the AP and lateral views [12]. All analysed publications reported a decrease in the talocalcaneal angle post-operatively (Figs. 5 and 6).

The unweighted MDs of the anteroposterior talocalcaneal angle for arthroereisis, osteotomy and combined operations respectively were (−6.81 degrees (95% CI: −6.85, −6.77); −11.73 degrees (95% CI: −11.79, −11.67); −9.50 degrees (95% CI: −9.73, −9.27)). The corresponding post-operative anteroposterior talocalcaneal angle of 22.72 degrees, 23.55 degrees and 16.50 degrees were found to be within that of the normal population’s mean (normal: 15 degrees to 25 degrees), indicative of a decrease in the extent of hindfoot valgus. Compared to subtalar arthroereisis, osteotomy demonstrated a significantly greater reduction in the AP Kite’s angle (*p* <.0001).

The unweighted MDs of the lateral talocalcaneal angle for arthroereisis, osteotomy and combined operations respectively were (−7.14 degrees (95% CI: −7.21, −7.07); −4.24 degrees (95% CI: −4.88, −3.60); −4.93 degrees (95% CI: −5.32, −4.54)). The corresponding post-operative lateral talocalcaneal angle of 29.79 degrees, 22.33 degrees and 35.47 degrees were found to be close to that of the normal population’s mean (normal: 25 degrees to 40 degrees) [80], indicative of a decrease in the extent of hindfoot valgus. Compared to osteotomy, arthroereisis demonstrated a significantly greater reduction in the lateral talocalcaneal angle (*p* <.0001).

**Fig. 7:**
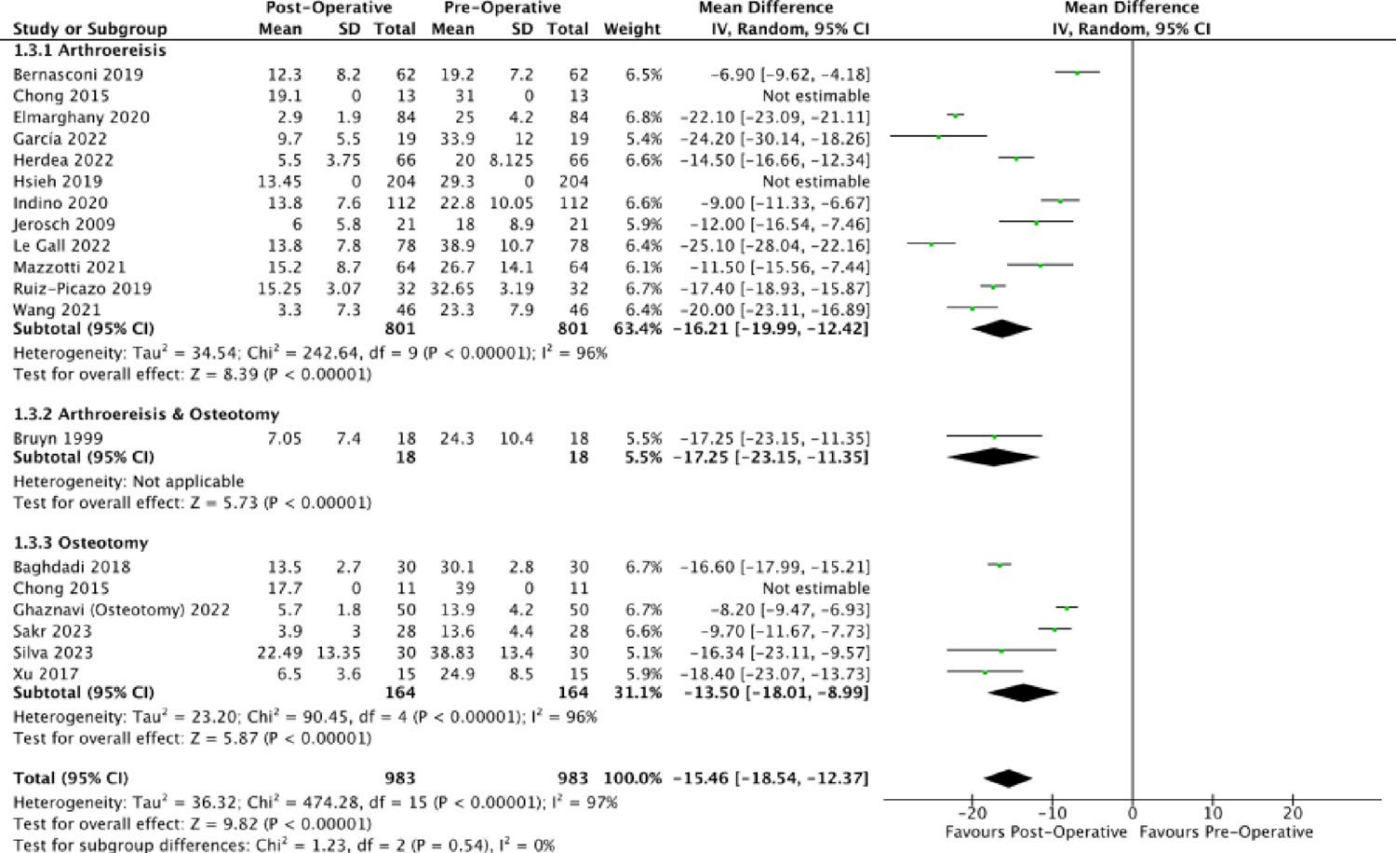
Forest plot comparing the pre- and post-operative outcomes of the talonavicular coverage angle

All analysed papers consistently reported on a decrease in talonavicular coverage angle with unweighted MDs of (−15.63 degrees (95% CI: −15.87, −15.39), −12.72 degrees (95% CI: −13.22, −12.22), −17.25 degrees (95% CI: −8.64, −5.86) for arthroereisis, osteotomy and combined operations (Fig. 7). The corresponding mean post-operative talonavicular coverage angles of 10.13 degrees, 10.27 degrees and 7.05 degrees indicate correction of the forefoot abduction to an acceptable limit within the normal population (normal: 15 degrees to 20 degrees) [81]. Compared to osteotomy, subtalar arthroereisis demonstrated a significantly greater reduction in talonavicular coverage angle (*p* <.0001).

**Fig. 8:**
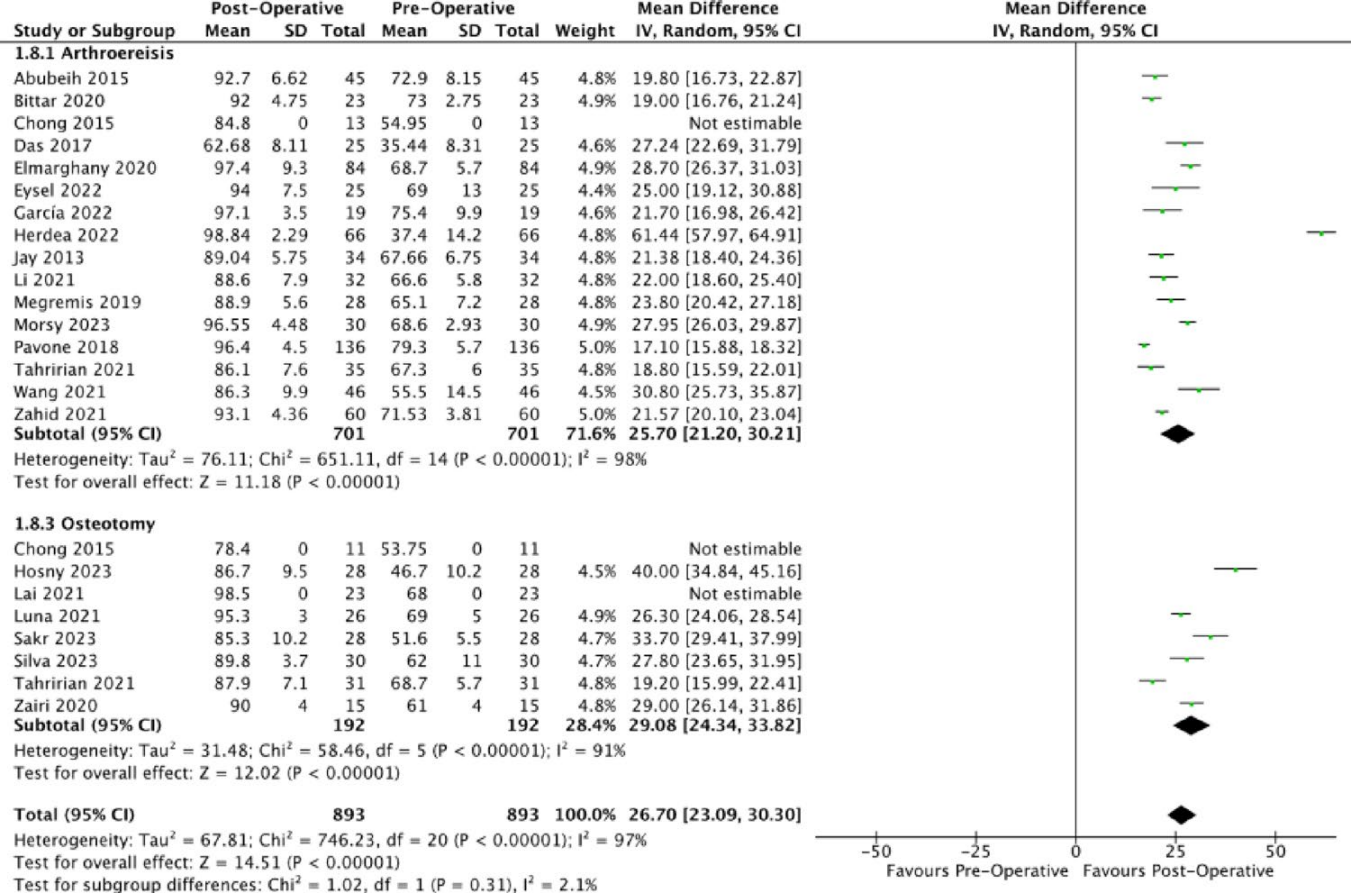
Forest plot comparing the pre- and post-operative outcomes of the AOFAS Ankle-Hindfoot score

All analysed publications similarly reported an improvement in post-operative outcomes, as evidenced by the increase in the total AOFAS score. The corresponding mean post-operative AOFAS scores were 92.56 and 89.00. The unweighted MDs were 26.37 (95% CI: 25.94, 26.80) and 29.18 (95% CI: 28.60, 29.77) for arthroereisis and osteotomy respectively (Fig. 8). However, the 2.81 difference between the unweighted MDs of arthroereisis and osteotomy did not fulfil the minimal clinically important difference (MCID) of 5.84 for AOFAS score [82].

In terms of the post-operative complications, a total of 420 feet (9.4%) reported complications in the 60 studies included. The detailed list of complications found in each study is listed in Table 2. Amongst the 46 studies which employed arthroereisis, the most common complication documented was persistent pain typically around the sinus tarsi region, reported in 107 feet (2.6%). Should conservative measures such as rest, application of a local ice bag, and nonsteroidal anti-inflammatory drugs (NSAIDs) fail to resolve the sinus tarsi pain, these patients would undergo premature implant removal or revision surgeries. For the 15 studies that investigated osteotomy, the most prevalent complication detailed were superficial and deep infections, reported in 20 feet (4.5%) and 4 feet (0.9%) respectively.

The NOS was applied to score and classify the quality of included studies (Table 4). 10 out of the 60 studies (16.7%) were classified as low risk of bias, 50 studies (83.3%) as moderate risk of bias and no studies as high risk of bias.

## Discussion

The principal finding of this study challenges the traditional assumption that osteotomy provides universally superior radiographic correction. Instead, our analysis reveals a nuanced landscape: while osteotomy yields superior correction of calcaneal pitch and hindfoot valgus on the anteroposterior view, subtalar arthroereisis demonstrates significantly greater correction of the lateral longitudinal arch and talonavicular coverage. Furthermore, both procedures resulted in comparable functional outcomes, with arthroereisis maintaining a more favourable safety profile.

Osteotomy was shown to have greater significant improvements for anteroposterior Meary’s angle (−12.71 vs. −9.82, *p* <.0001), anteroposterior Kite’s angle (−11.73 vs. −6.81, *p* <.0001) and calcaneal pitch, (11.14 vs. 4.05, *p* <.0001). This confirms that osteotomy is biomechanically superior in restoring the structural inclination of the calcaneus and correcting hindfoot valgus in the coronal plane. Arthroereisis had greater improvement in the lateral Meary’s angle (−11.71 vs. −10.08, *p* <.0001), lateral Kite’s angle (−7.14 vs. −4.24, *p* <.0001) and talonavicular coverage angle (−15.63 vs. −12.72, *p* <.0001). This suggests that the sinus tarsi implant is particularly effective at blocking the anterior and plantar translation of the talus, thereby forcefully reducing forefoot abduction and restoring the medial longitudinal arch height on the lateral view. This finding aligns with the mechanism of arthroereisis, which acts as a pivot to block talotarsal hyperpronation, effectively stabilizing the transverse tarsal joint during dynamic loading [39]. 

However, clinical and functional outcomes measured by the AOFAS score do not significantly differ between subtalar arthroereisis and osteotomy, as both procedures achieve comparable improvements and the difference between their unweighted MDs do not exceed the MCID threshold. Overall, the results of this SRMA are consistent with those of Chong et al. and Tahririan et al., both of which demonstrated significant clinical and radiographic improvements with subtalar arthroereisis and lateral calcaneal lengthening and hence reinforcing subtalar arthroereisis as a viable, less-invasive alternative to calcaneal osteotomy for managing paediatric flexible flatfoot.

Undoubtedly, the selection of appropriate interventions for the correction of paediatric pes planus deformity is multifactorial and complex. There is no one consensus on an algorithm, although an article by Harris et al. [83] has sought to outline a treatment protocol. The main objective of any deformity correction procedure should be to achieve the best correction whilst maximising the preservation of joint function. Subtalar arthroereisis has been gaining favour owing to its minimal invasiveness, reduced morbidity and rapid recovery time. Subtalar arthroereisis is also suitable as an outpatient surgery, with smaller incisions and earlier weight-bearing capacity. Unlike earlier studies that reported high failure rates and considerable complications associated with older implant designs [7], this study shows that the overall complication rate for subtalar arthroereisis is lower than that of osteotomy, with rates of 9.2% compared to 10.5%, though this was not statistically significant (*p* =.39).

Although subtalar arthroereisis is easily reversible by removal of the implant, reservations stem from the potential for associated arthritis, which may arise from poor implant placement that restricts subtalar joint movement [5]. Inadequate implant sizing can also lead to poor correction, and increased pain in the sinus tarsi and implant sites. However, with meticulous training and expertise in performing subtalar arthroereisis, it is possible to minimise the risk of implant failure and achieve similar success rates and patient satisfaction comparable to osteotomies, which are traditionally favoured in yielding more predictable long-term corrections. In surgical practice, it is also crucial to integrate different techniques to address various musculoskeletal forces causing deformity. The single study by Bruyn et al., [63] demonstrated how the combination of Evans calcaneal osteotomy and STA-Peg arthroereisis was successfully utilised alongside concomitant soft tissue techniques and arthrodesis to treat severe flexible pes planovalgus deformity.

In this review, there was an inadequacy of studies for further sub-group analysis. However, several studies offered insights into specific variables that could contribute to more optimal outcomes for arthroereisis and osteotomy procedures, such as body mass index (BMI), surgical techniques, type of implants and the method of fixation. For instance, in a retrospective cohort study conducted by Monestier et al., [84] higher BMI was associated with more pain and poorer radiographic correction following subtalar arthroereisis. Similarly, Hsieh et al. [36] observed obese children had a significantly increased risk of implant extrusion after subtalar arthroereisis, in a total of 86 feet out of 204 feet (42.2%). Hence, taking into account a patient’s BMI allows for a more precise assessment of the benefit-to-risk ratio, leading to the selection of better candidates for surgical intervention.

It was initially hypothesised that different surgical techniques in arthroereisis might account for outcome heterogeneity. However, the study by Memeo et al. [48] involving 402 feet found no significant differences in clinical or radiographic outcomes between exosinotarsal arthroereisis with screw implants and endosinotarsal correction with spacer implants, suggesting that the choice of technique could be based on individual surgeon preference. In our review, the spacer implant was used in 1856 feet, while the screw implant was used in 2224 feet. Similar to the findings of a study by Zahid et al., [62] which compared spacer and screw implants over a 60-feet cohort, this SRMA found that screw implants were associated with lower rates of persistent pain (38 vs. 69, 1.7% vs. 3.7%, *p* <.0001) (Table 2). These outcomes suggest that screw implants may be a preferable option due to their similar clinical and radiological performance, reduced complication rates, and cost-effectiveness.

Meanwhile, in terms of calcaneal lengthening osteotomy, Abhishek et al. [75] strongly advocated for K-wire over plate fixation. While both fixation methods yielded comparable radiographic and functional results in this study of 102 feet, K-wire fixation was associated with reduced cost and a 17.7-fold increased risk of reoperations for painful hardware. Of interest, a retrospective comparative study performed by Moraleda et al. [85] concluded that both calcaneo-cuboid-cuneiform osteotomy and calcaneal lengthening osteotomy achieved similar clinical and radiographic results, but calcaneal lengthening osteotomy led to greater improvement of the alignment of the navicular and talar head, despite being associated with more frequent and more severe complications. Since the cuboid and navicular function as a unit, calcaneal lengthening induces a medial and plantar translocation of the navicular relative to the talar head. However, surgeons should keep in mind that calcaneal lengthening alone may be unable to completely reduce the talocalcaneonavicular joint complex in patients with severe deformities, and additional procedures may be required.

Key limitations of this review are outlined below. First, significant diversity in study designs, patient populations, and interventions contributed to the heterogeneity of results, which precluded subgroup analysis. Additionally, although we included all type of arthroereisis and osteotomies, there was an inadequacy of studies for further sub-group analysis. Second, the mean follow-up duration was relatively short, with 49.7 months for the arthroereisis studies and 27.8 months for the osteotomy studies. This limits the conclusion to be drawn on the long-term outcomes and efficacy of both interventions into adulthood. Third, the current literature is in its infancy and high-quality prospective data is lacking. The evidence base is predominantly retrospective (Level III and IV), with 83.3% of included studies classified as having a moderate risk of bias. This introduces potential selection bias; specifically, the decision to perform osteotomy versus arthroereisis was often based on surgeon preference or deformity severity rather than randomization. Thus far, only one study conducted by Tahiririan et al. ran a prospective head-to-head RCT comparing the functional and radiographic outcomes of subtalar arthroereisis versus lateral calcaneal osteotomy. Although subtalar arthroereisis is recognised as a minimally invasive surgical intervention for pes planus, further high-quality RCTs are warranted to evaluate the long-term efficacy of its corrective outcomes and address persistent concerns regarding utilisation of this procedure, particularly when compared to traditional procedures such as calcaneal osteotomy.

## Conclusion

This systematic review and meta-analysis demonstrates that osteotomy and subtalar arthroereisis possess distinct radiographic strengths in the management of paediatric idiopathic flexible flatfoot. While osteotomy provides superior restoration of calcaneal pitch and correction of hindfoot valgus in the coronal plane, subtalar arthroereisis offers significantly greater correction of the lateral longitudinal arch and forefoot abduction. Critically, these radiographic differences do not translate into a clinically significant divergence in functional outcomes, with both procedures yielding equivalent AOFAS scores. Given the comparable efficacy, lower risk of deep infection, and minimally invasive nature of subtalar arthroereisis, it may be considered a primary surgical intervention, with osteotomy reserved for severe structural calcaneal deformities or revision cases. Future high-quality, prospective randomised trials are essential to validate these findings and refine treatment algorithms.

## Data Availability

Data is provided within the manuscript or supplementary information files.
